# Supplementary material for: COVID-19 among Czech Dentistry Students: Higher Vaccination and Lower Prevalence Compared to General Population Counterparts
Source: Vaccines (Basel). 2022 Nov 14;10(11):1927. doi: 10.3390/vaccines10111927 (PMC9695306; doi:10.3390/vaccines10111927)
Supplement: Supplementary file 1 [file vaccines-10-01927-s001.zip › vaccines-1999215-supplementary.pdf]

STROBE Statement—Checklist of items that should be included in reports of ***cross-sectional studies***

	Item No	Recommendation
**Title and abstract**	1	(*a*) Indicate the study's design with a commonly used term in the title or the abstract<br>*Cross-sectional study as stated in the Abstract on page 1 and Methods on pages 2-4.*
		(*b*) Provide in the abstract an informative and balanced summary of what was done and what was found<br>*Provided in Abstract on page 1.*
**Introduction**		
Background/rationale	2	Explain the scientific background and rationale for the investigation being reported<br>*Included in Introduction on pages 1-2.*
Objectives	3	State specific objectives, including any prespecified hypotheses<br>*Included in Introduction on page 2.*
**Methods**		
Study design	4	Present key elements of study design early in the paper<br>*Included in Methods on pages 2-4.*
Setting	5	Describe the setting, locations, and relevant dates, including periods of recruitment, exposure, follow-up, and data collection<br>*Included in Methods on pages 2-4.*
Participants	6	(*a*) Give the eligibility criteria, and the sources and methods of selection of participants<br>*Included in Methods on pages 2-3.*
Variables	7	Clearly define all outcomes, exposures, predictors, potential confounders, and effect modifiers. Give diagnostic criteria, if applicable<br>*Included in Methods on pages 2-3.*
Data sources/ measurement	8*	For each variable of interest, give sources of data and details of methods of assessment (measurement). Describe comparability of assessment methods if there is more than one group<br>*Included in Methods on pages 2-4.*
Bias	9	Describe any efforts to address potential sources of bias<br>*Included in Discussion on page 16.*
Study size	10	*Included in Methods on pages 2-3.*
Quantitative variables	11	Explain how quantitative variables were handled in the analyses. If applicable, describe which groupings were chosen and why<br>*Included in Methods on pages 2-4.*
Statistical methods	12	(*a*) Describe all statistical methods, including those used to control for confounding<br>*Included in Methods on pages 2-4.*
		(*b*) Describe any methods used to examine subgroups and interactions<br>*Included in Methods on pages 2-4.*
		(*c*) Explain how missing data were addressed<br>*Included in Methods on page 3 and Results on page 4.*
		(*d*) If applicable, describe analytical methods taking account of sampling strategy<br>*Included in Methods on pages 2-4.*
		(*e*) Describe any sensitivity analyses<br>*Not applicable.*
**Results**		
Participants	13*	(a) Report numbers of individuals at each stage of study—eg numbers potentially eligible, examined for eligibility, confirmed eligible, included in the study, completing follow-up, and analysed

		Included in Results on page 4.
		(b) Give reasons for non-participation at each stage<br>*Not applicable.*
		(c) Consider use of a flow diagram<br>*Not required.*
Descriptive data	14*	(a) Give characteristics of study participants (eg demographic, clinical, social) and information on exposures and potential confounders<br>*Included in Results on pages 4-5.*
		(b) Indicate number of participants with missing data for each variable of interest<br>*Included in Results on page 4.*
Outcome data	15*	Report numbers of outcome events or summary measures<br>*Included in Results on pages 4-12.*
Main results	16	(*a*) Give unadjusted estimates and, if applicable, confounder-adjusted estimates and their precision (eg, 95% confidence interval). Make clear which confounders were adjusted for and why they were included<br>*Included in Results on pages 4-12.*
		(*b*) Report category boundaries when continuous variables were categorized<br>*Included in Results on pages 4-12.*
		(*c*) If relevant, consider translating estimates of relative risk into absolute risk for a meaningful time period<br>*Not applicable.*
Other analyses	17	Report other analyses done—eg analyses of subgroups and interactions, and sensitivity analyses<br>*Included in Results on pages 11-12.*
**Discussion**		
Key results	18	Summarise key results with reference to study objectives<br>*Included in Discussion on pages 12-15.*
Limitations	19	Discuss limitations of the study, taking into account sources of potential bias or imprecision. Discuss both direction and magnitude of any potential bias<br>*Included in Discussion on page 15.*
Interpretation	20	Give a cautious overall interpretation of results considering objectives, limitations, multiplicity of analyses, results from similar studies, and other relevant evidence<br>*Included in Discussion on pages 12-15.*
Generalisability	21	Discuss the generalisability (external validity) of the study results<br>*Included in Discussion on pages 12-15.*
**Other information**		
Funding	22	Give the source of funding and the role of the funders for the present study and, if applicable, for the original study on which the present article is based<br>*Included on page 15.*

*Give information separately for exposed and unexposed groups.

Note: An Explanation and Elaboration article discusses each checklist item and gives methodological background and published examples of transparent reporting. The STROBE checklist is best used in conjunction with this article (freely available on the Web sites of PLoS Medicine at http://www.plosmedicine.org/, Annals of Internal Medicine at http://www.annals.org/, and Epidemiology at http://www.epidem.com/). Information on the STROBE Initiative is available at www.strobe-statement.org.
Page numbers refer to the version of the document in Microsoft Word format.
